# Patient satisfaction in an “open-door” acute inpatient psychiatric unit

**DOI:** 10.1192/j.eurpsy.2023.1913

**Published:** 2023-07-19

**Authors:** M. Campillo, L. Rius, S. Garcia, M. Olivero, G. Sanchez Tomico, M. Martinez Garcia, I. Garcia Velasco, C. Monserrat, A. Pratdesava, R. Sanchez

**Affiliations:** 1 Psychiatry, Parc de Salut Mar, Santa Coloma Gramenet, Spain

## Abstract

**Introduction:**

Traditionally, psychiatric wards had established a “locked door” policy but secluded conditions may increase patient’s discomfort1 that could affect the perception of health quality of care2. Recently, the “open-door” policy is being adopted in several European countries but its impact on patient satisfaction remains unknown (Schreiber, LK. BMC Psychiatry. 2019 May 14;19(1):149). Since 2019 our psychiatric hospital has implemented the open-door policy.

**Objectives:**

The aim of this study is to investigate the impact of the “open-door” policy on patient satisfaction during their stay in the acute inpatient unit of our psychiatric hospital.

**Methods:**

This is an observational study. Prior to the implementation of the open door policy 31 patient satisfaction data was collected between October 2018 to April 2019 and it was also assessed with 31 subjects between July to October 2019, after the implementation of the open “door-policy”. The inclusion criteria were being >18 years old, reading Spanish correctly and with a length of stay >72 hours. The patients with dementia disorder and intellectual disability where excluded from the study. We used the Satispsy-22-E scale, a self-administered questionnaire (Frías, V., et al. 2018. Psychiatry Res. Oct;268:8-14). It assesses patient’s experience of hospitalization through 22 items distributed into 6 dimensions. The score range is from 0 to 100. Differences in Satispsy-22-E scores were analysed by applying ANOVA using the IBM-SPSS (v. 25).

**Results:**

Total scores in Satispsy-22 are provided in Figure 1. We found that patient satisfaction was increased in the dimensions of “personal experience” and “food” (p<0.05). No significant differences were found in staff, quality of care, information, activity dimensions and Total score (Table 2).

**Table:** 

**Dimension**	**F-Test**	**Statistic Value**
**Staff**	1.402	p=0.241
**Quality of Care**	841	p=0.362
**Personal Experience**	4.071	p=0.048*
**Information**	656	p=0.420
**Activity**	434	p=0.512
**Food**	4.507	p=0.037*
**TOTAL**	3.645	p=0.61

**Image:**

**Figure:**
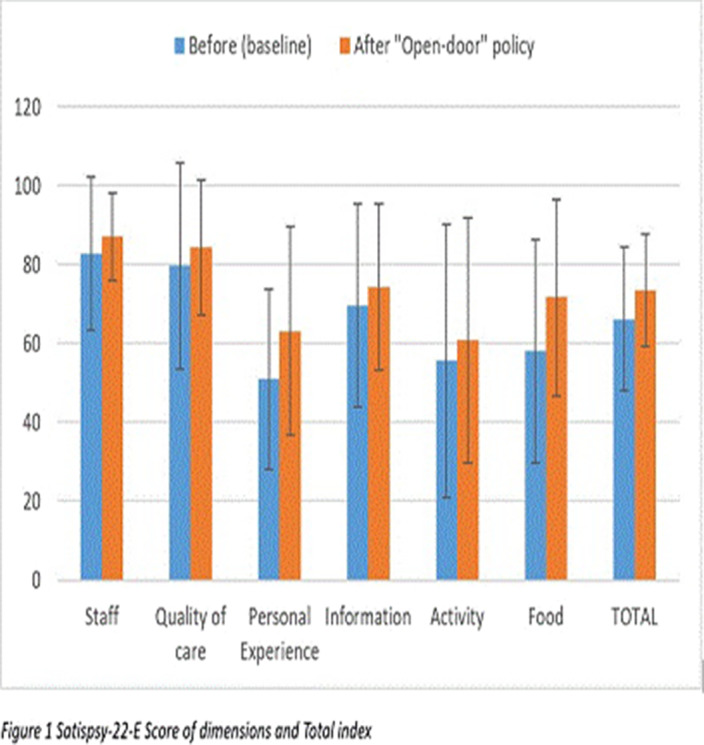

**Conclusions:**

Our results provide preliminary evidence indicating that the open-door policy could have a positive impact on patient satisfaction, especially in relation to the personal experience on an acute inpatient psychiatric unit.

**Disclosure of Interest:**

None Declared

